# The Effectiveness of Ketamine in Pediatric Acute Deafferentation Pain after Spinal Cord Injury

**DOI:** 10.1155/2020/8835292

**Published:** 2020-10-19

**Authors:** Devina G. Shiwlochan, Misty Shah, Khushboo Baldev, Donna-Ann Thomas, Maxime Debrosse

**Affiliations:** Department of Anesthesiology, Yale School of Medicine, New Haven, Connecticut, USA

## Abstract

Deafferentation pain and allodynia commonly occur after spinal cord trauma, but its treatment is often challenging. The literature on effective therapies for pediatric deafferentation pain, especially in the setting of spinal cord injury, is scarce. We report the case of a 12-year-old patient with acute allodynia after a gunshot injury to the spine. The pain was refractory to multiple analgesics, but resolved with ketamine, which also improved the patient's physical function and quality of life, a trend that continued many months after the injury. We suggest that early initiation of ketamine may be effective for acute pediatric deafferentation pain secondary to spinal cord injury, as well as preventing chronic pain states in that population.

## 1. Introduction

Deafferentation pain is thought to be the result of loss of normal input from primary sensory neurons—typically after a peripheral nerve injury [1, 2]. Allodynia, which is defined as pain due to nonnoxious stimuli, is a common manifestation of deafferentation pain, whereby increased N-methyl-D-aspartate (NMDA) and glutamate receptor activation and decreased inhibition are believed to occur [3]. Neuropathic phenomena after spinal cord injury (SCI) are challenging to manage and notoriously resistant to opioids [4]. Moreover, the literature on the use of ketamine, an NMDA antagonist, in pediatric SCI-induced deafferentation pain is scant. We present the effectiveness of subanesthetic ketamine in a 12-year-old child with acute deafferentation pain and refractory allodynia secondary to traumatic SCI.

## 2. Case Description

**Figure 1:**
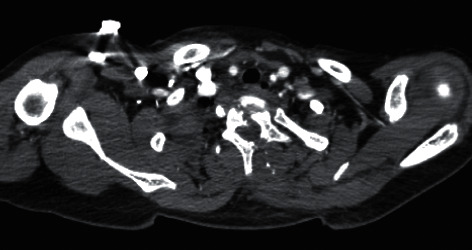
Axial slice of the patient's preoperative chest CT showing three-column fracture at T1 and T2 levels. The hypodense diagonal path bisecting the vertebra illustrates shattering of the vertebral body and posterior elements, presumably from the bullet's impact and trajectory.

This is an otherwise healthy 12-year-old, 33-kilogram male who was transferred to a pediatric intensive care unit (PICU) in the United States from another hospital 3 days after sustaining a stray gunshot injury to the thoracic spine. Chest CT (computed tomography) scan showed T1 and T2 fractures and spinal cord transection (Figure 1).

On arrival to the PICU, the Pediatric Pain service was consulted. The patient reported forearm pain, rated 8/10 on the Numerical Rating Scale (NRS), described as burning and shock-like, and worsened by touch. At that time, he was on intravenous (IV) injections of hydromorphone 0.3 mg q4 h as needed for pain, as well as standing doses of gabapentin 300 mg q8 h, ketorolac 15 mg q6 h, and acetaminophen 650 mg q4 h. This regimen reduced the pain to 7/10. Examination revealed a significantly distressed child with normal vital signs, normal heart rhythm and sounds, and clear lungs. Neurologically, he was fully alert and oriented. Cranial nerve (CN) testing was intact, except for CN XI, which was limited due to pain. He had 0/5 strength in his lower extremities. Deep tendon reflex testing was deferred as he reported that it sent “shocks” to his spine on prior exams. He wiggled his fingers on command, but did not want to move his upper extremities from fear of pain. Light touch sensation was absent below the T3 dermatome, and above that level, he had exquisite allodynia in both forearms, worse on the right side, such that the exam was stopped on his request. Laboratory studies were within normal limits.

He was started on hydromorphone IV nurse-controlled analgesia (hNCA) without a basal rate, 0.1 mg demand dose with 15-minute lockout interval, and rescue dose of 0.3 mg q4 h as needed for breakthrough pain (for a maximum dose of 1.9 mg over a 4-hour period). An NCA modality was chosen over PCA (patient-controlled analgesia) because his hand allodynia was so excruciating that he was unable to push the activation button of the PCA pump. His allodynia persisted, so the hNCA demand and gabapentin doses were doubled. The Neurosurgery team was consulted, and on hospital day #2, the patient was taken to the operating room (OR), where he underwent general anesthesia with an endotracheal intubation. Intraoperative pain was controlled with IV fentanyl (50 mcg for induction and 50 mcg prior to incision) and nonopioid adjuvants consisting of acetaminophen 600 mg and ketamine which was initially bolused 20 mg (0.6 mg/kg) and an infusion maintained at 0.25 mcg/kg/min. The surgical team performed a C6 to T4 posterior spinal instrumentation and fusion, T2 laminectomy and fracture reduction, dural repair, and complex closure of the bullet exit wound. Intraoperative findings included bony fragments in the paraspinal muscles and a highly contused spinal cord. He was extubated in the OR in stable condition and was brought to the PICU, where a lumbar CSF (cerebrospinal fluid) drain was placed and was removed 2 days later.

**Figure 2:**
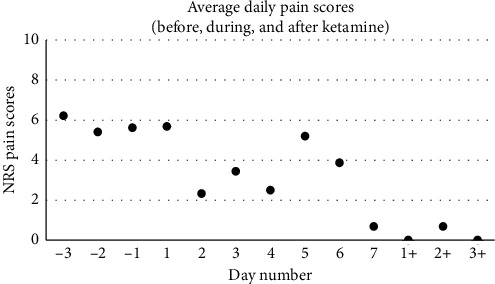
Average daily NRS pain scores before, during, and after ketamine. Day number −3 corresponds to 3 days preceding ketamine initiation, and day number 1 is the first day of the ketamine treatment. Of note, the patient's spinal surgery occurred on day −1, and day 1 also represents postoperative day 1 (POD1). Day numbers +1 to +3 refer to the days following ketamine discontinuation. The graph shows a downtrend in average pain scores.

**Figure 3:**
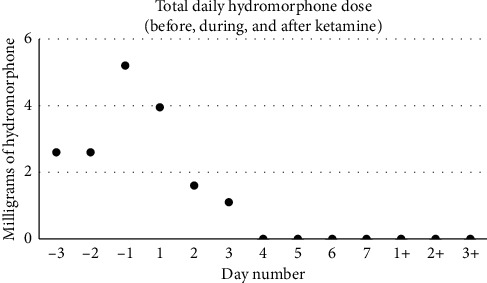
Total daily dose of hydromorphone in milligrams. Day number −3 corresponds to 3 days preceding ketamine initiation, and day number 1 is the first day of the ketamine treatment. Of note, the patient's spinal surgery occurred on day −1, and day 1 also represents postoperative day 1 (POD1). Day numbers +1 to +3 refer to the days following ketamine discontinuation. The graph shows a downtrend in daily hydromorphone dose. From ketamine day number 4, the patient did not receive any hydromorphone.

On postoperative day #1 (POD1), despite resuming his preoperative analgesics, the allodynia resurfaced, and he reported severe pruritus. The hNCA was thus discontinued, and a ketamine infusion was started at 5 mg/hr. The order for breakthrough-pain hydromorphone remained active. Early in the morning of POD2 (corresponding to ketamine day #2, or KD2), the allodynia worsened, and the ketamine rate was increased to 0.24 mg/kg/hr. The allodynia subsequently improved such that he no longer required doses of hydromorphone (Figure 2). Specifically, his average NRS pain scores were as follows: 5.7 during the 3 days preceding the ketamine infusion, 3.4 while on ketamine, and 0.2 over the 3 days following ketamine discontinuation. Total daily hydromorphone requirements steadily decreased (Figure 3). More precisely, his average daily hydromorphone requirements were 3.5 mg preketamine, 0.95 mg on ketamine, and 0 mg postketamine, corresponding to daily morphine-milligram equivalents (MME) of 17.3, 4.8, and 0, respectively. On KD3, he became able to comfortably touch objects (namely, using his cell phone). To further optimize his analgesia, adjuvant analgesics were started, namely, oral clonidine 0.1 mg on the night of KD2, duloxetine, and pregabalin on KD4. Gabapentin was discontinued due to concerns of poor efficacy despite the high dose, which led to rotation to pregabalin. Of note, he had moderate spasms postoperatively and received a daily average of 2 doses of oral diazepam until POD6. His pruritus gradually improved. He did not report psychotomimetic effects while on ketamine, but he had one emesis episode (on KD5), which resolved with ondansetron. From KD4 onwards, he tolerated physical therapy exercises. His quality of life improved as well; he reported better sleep, feeling hopeful about his recovery, and he started playing video games, a hobby he has normally enjoyed until the gunshot injury. He was transferred from the PICU to the regular nursing floor on KD5, and ketamine was tapered down by 0.1 mg/kg/hr starting on POD5 and discontinued on POD7. He was discharged to a rehabilitation facility on POD15. The patient and his father were contacted 8 months after discharge for follow-up, and the patient reported 0/10 pain. He continued physical therapy, and provider notes reported increased muscle strength from 2/5 to 3/5 in his lower extremities. He has continued schoolwork and to play video games during weekends. The patient and his father were both pleased with the inpatient pain treatment.

## 3. Discussion

This case describes the beneficial effects of ketamine in a pediatric patient with acute deafferentation pain after an ASIA A spinal cord injury. After initiation of a ketamine infusion at 0.24 mg/kg/hr, significant improvements were noted in his pain, function, and quality of life. Strikingly, within a few days of starting the treatment, he no longer required opioids. However, some points merit discussion. Studies have showed effective reduction in postoperative pain when ketamine is initiated intraoperatively. However, the degree to which intraoperative ketamine influences postoperative analgesia is unknown. Variable intraoperative boluses and infusion rates have been used (bolus rates from 0.15 to 0.5 mg/kg and infusion rates from 2 to 10 mcg/kg/min). It has also been long known that ketamine can help decrease the postoperative opioid requirement in patients undergoing spine surgery and those with CRPS. However, its efficacy in opioid naïve patients is less well-known. Porter et al. gathered randomized trials that studied the effect of ketamine in spine surgeries and demonstrated significant decrease in postoperative opioid requirement, but Loftus et al. noted that this was significant for only chronic pain patients who were on >40 mg/day of MME [5]. The relative increase in average pain scores on KD5 and KD6 in Figure 2 was probably due to positional changes during his transport from the PICU to the regular floor, as well as escalation of his physical therapy. More importantly, he did not require any opioids on those days, and his gains in functionality and quality of life persisted. Still, a limitation of our case is the possibility that the patient had episodes of higher pain scores than those reflected in the electronic record, from which the graph values were extracted. It is thus conceivable that the charted pain scores underestimated actual values, which may have overestimated the ketamine benefit. Another limitation is that we are unable to determine the extent to which the surgical decompression of his spine contributed to his postoperative pain.. Lastly, one may wonder if the pain reduction was a mere improvement in the patient's incisional pain. However, as shown Figure 2, the average daily NRS scores in the days before and immediately after surgery were quite similar, and after the ketamine, NRS scores were decreased to below presurgical levels. This graphical observation led us to conclude that the ketamine impacted the presurgical SCI pain, as opposed to just incisional pain, although it likely helped both phenomena.

We can infer that early ketamine initiation helped prevent the development of chronic pain in our patient, as the pain remained resolved even 8 months after his hospital discharge. Moreover, he continued to achieve strength gains through physical therapy, as well as enjoying his hobby of playing video games. Sleigh et al. have suggested that early, short-term, NMDA receptor antagonism during the acute pain phase induces prolonged downregulation of central hyperexcitability triggered by nerve injury, and that a “wind-down” phenomenon may explain long-term pain relief (5 h). In terms of side effects, ketamine has been stated to cause CNS excitation, sedation, visual disturbances, hemodynamic instability, nausea, and elevated LFTs (liver function tests). . In fact ketamine-induced liver injury (KILI) has been reported in patients who had repeated ketamine infusions lasting about 16 days, though dosages vary. Zhu et al. noted two case reports where patients received ketamine infusions (normal preoperative liver function tests) with as large as a 3-year gap and still experienced KILI within 22–48 hours after ketamine initiation, even though LFTs returned to normal after the cessation of ketamine [6]. No preoperative LFTs were noted for our patient. However, on POD 5, LFTs were drawn for unknown reason and noted for mildly elevated ALT 138. No changes to medications were made due to these results. The vomiting episode experienced by our patient on KD5 might have been due to ketamine, but duloxetine and pregabalin, both of which were started 24 hours before the emesis, might have played a role. Porter noted in patients who had total knee arthroplasty and concluded that ketamine use was correlated with a reduction in postoperative nausea and vomiting [5]. Ketamine was associated with a reduction in sedation. Using the Richmond Agitation Sedation Scale (RASS), it was seen that, while on hNCA, the patients' scored from −1 to −2, whereas while on ketamine, RASS scores remained at 0. The absence of psychotomimetic effects in our patient was likelya result of diazepam doses he received for spasms combined with the dose-dependent nature of those side effects.

Studies in adult subjects have described ketamine's usefulness in combination with adjuvants [7–10], which may lead one to wonder whether concurrent postoperative analgesics (duloxetine, pregabalin, and clonidine) contributed to our patient's analgesia. But given their timing, we believe ketamine was the main driver of his pain outcomes. More precisely, it is unlikely that duloxetine helped, given its known delayed onset of action. Moreover, clonidine and pregabalin were started after the patient's pain, function, and quality of life were already improved. Therefore, while ketamine likely caused the early-onset analgesia, it is possible that the adjuvants helped maintain it, while also contributing to sleep and mood benefits.

In conclusion, early initiation of ketamine in pediatric SCI patients may improve deafferentation pain, function, quality of life, as well as potentially preventing chronic SCI pain. Importantly, the opioid-sparing benefit of ketamine may enhance patient safety. Further studies will help expand the role of ketamine in pediatric pain secondary to spine trauma.

## Data Availability

The data were extracted from retrospective review of the patient's chart.
